# Cyanobacteria-shrimp colonies in the Mariana Islands

**DOI:** 10.1007/s10452-021-09837-6

**Published:** 2021-02-27

**Authors:** Christopher A. Leber, Andres Joshua Reyes, Jason S. Biggs, William H. Gerwick

**Affiliations:** Center for Marine Biotechnology and Biomedicine, Scripps Institution of Oceanography, University of California, San Diego, La Jolla, CA 92093, USA; University of Guam Marine Laboratory, UOG Station, Mangilao, GU 96923, USA; University of Guam Marine Laboratory, UOG Station, Mangilao, GU 96923, USA; Skaggs School of Pharmacy and Pharmaceutical Sciences, University of California, San Diego, La Jolla, CA 92093, USA; Center for Marine Biotechnology and Biomedicine, Scripps Institution of Oceanography, University of California, San Diego, La Jolla, CA 92093, USA

**Keywords:** *Moorena bouillonii*, *Alpheus frontalis*, Symbiosis, Cyanobacteria, Snapping shrimp, Coral reefs

## Abstract

Cyanobacteria have multifaceted ecological roles on coral reefs. *Moorena bouillonii*, a chemically rich filamentous cyanobacterium, has been characterized as a pathogenic organism with an unusual ability to overgrow gorgonian corals, but little has been done to study its general growth habits or its unique association with the snapping shrimp *Alpheus frontalis*. Quantitative benthic surveys, and field and photographic observations were utilized to develop a better understanding of the ecology of these species, while growth experiments and nutrient analysis were performed to examine how this cyanobacterium may be benefiting from its shrimp symbiont. Colonies of *M. bouillonii* and *A. frontalis* displayed considerable habitat specificity in terms of occupied substrate. Although found to vary in abundance and density across survey sites and transects, *M. bouillonii* was consistently found to be thriving with *A. frontalis* within interstitial spaces on the reef. Removal of *A. frontalis* from cyanobacterial colonies in a laboratory experiment altered *M. bouillonii* pigmentation, whereas cyanobacteria-shrimp colonies in the field exhibited elevated nutrient levels compared to the surrounding seawater.

## Introduction

Cyanobacteria, also known as blue-green algae, represent a wide array of organisms that operate in a diversity of roles in marine ecosystems. In addition to producing oxygen via photosynthesis, some cyanobacteria can also fix nitrogen; these capabilities make cyanobacteria important primary producers in many marine systems, especially in oligotrophic open ocean environments (Carpenter, Subramaniam and Capone 2004). Conversely, cyanobacteria, often empowered by anthropogenic perturbations, can disrupt aquatic ecosystems by forming sustained and deleterious blooms (CyanoHABs) that produce toxins and can cause localized hypoxia (Paerl and Paul 2012). In the context of coral reef ecosystems, a similar dichotomy can be identified. In addition to CyanoHABs occurring in and around coral reefs, cyanobacteria have been implicated in coral diseases, such as black band disease and grey-patch disease (Frias-Lopez et al. 2003; Sweet et al. 2019). Cyanobacteria also contribute positively to coral reef ecosystems, serving as habitat and sources of food (Cruz-Rivera and Paul 2002, 2006), and engaging in specialized symbioses, including with sponges (Schorn et al 2019) and shrimp (Banner and Banner 1982).

**Fig. 1:**
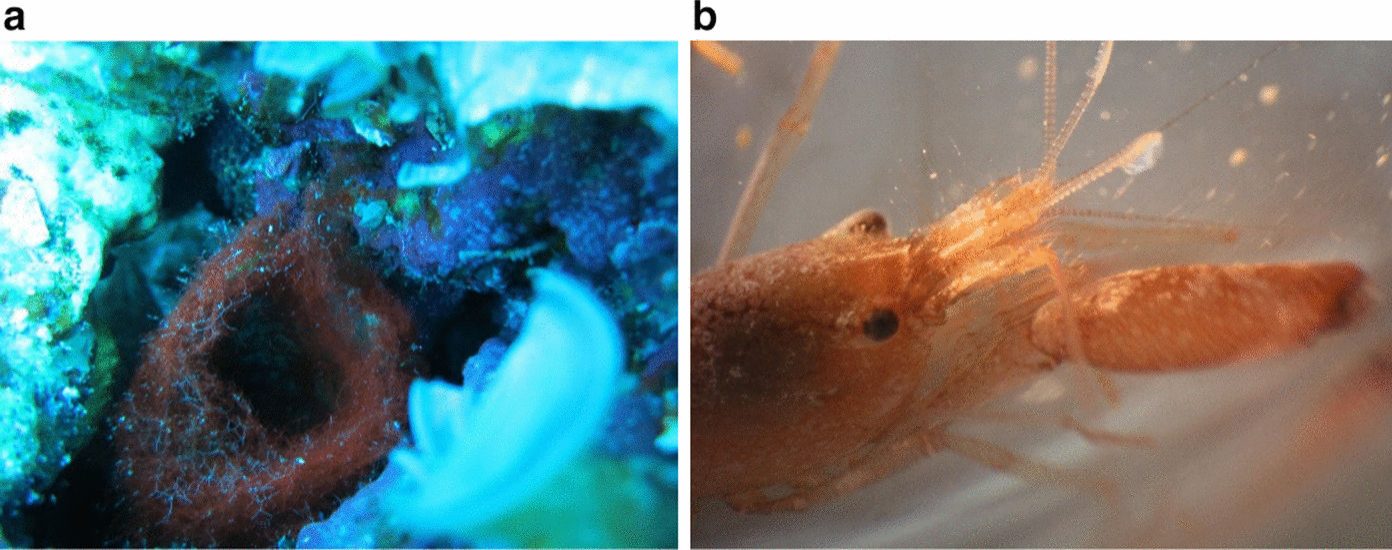
*Moorea bouillonii* and *Alpheus frontalis.* **a** A woven tube of the filamentous cyanobacterium *M. bouillonii* and **b** its shrimp symbiont *A. frontalis*

One intriguing interaction between a cyanobacterium and a tropical reef organism that was serendipitously observed through drug discovery efforts in the Tropical Western Pacific (Tan, Márquez and Gerwick 2002) is the association between the filamentous cyanobacterium *Moorena bouillonii* (Hoffmann and Demoulin) (Engene and Tronholm 2019) (Oscillatoriaceae) and a symbiotic snapping shrimp *Alpheus frontalis* H. Milne Edwards 1837 (Alpheidae) (Fig. 1). *A. frontalis* was first described in 1837 (Milne-Edwards 1837), and reports of its association with *M. bouillonii* date back to as early as 1880 (Richters 1880), when two shrimp were collected from Mauritius in woven tubes of cyanobacteria, which at the time were identified as *Oscillatoria* sp. *A. frontalis* weaves *M. bouillonii* filaments into labyrinthine shelters consisting of interconnected tunnels and chambers (see Supplementary Video S1). *Alpheus frontalis* (often collected with *M. bouillonii*) has been documented in various publications, catalogs of crustacean taxonomy, and collection records, suggesting that this partnership extends from the eastern African coast and the Red Sea, across the Indian Ocean, and throughout the Tropical Western Pacific, bounded by Australia, Southern Japan, and French Polynesia (Richters 1880; Fishelson 1966; Nomura et al. 1996; Poupin 1998; Simões, Apel and Jones 2001; Davie and Short 2001).

While much of the scholarly effort directed towards *A. frontalis* has focused on its taxonomic classification and distribution, research on *M. bouillonii* has largely centered on its rich secondary metabolite chemistry relevant to human health. In fact, the entire genus *Moorena* (previously *Moorea,* formerly a part of *Lyngbya*) has been identified as a prolific source of biologically active compounds (Leão et al. 2017; Tronholm and Engene 2019). Since its initial description in 1991 (Hoffmann and Demoulin 1991), more than 60 unique natural products have been discovered from *M. bouillonii* (according to the MarinLit database [https://pubs.rsc.org/marinlit/] and SciFinder [https://scifinder.cas.org/]), including the apratoxins, a family of exquisitely potent cancer cell cytotoxins (Luesch et al. 2001). In terms of driving ecological interactions among reef organisms, metabolites isolated from the closely related *M. producens* (previously *Lyngbya majuscula*) have been shown to be strong herbivore feeding deterrents (Nagle and Paul 1999); this has led to speculation that *M. bouillonii* may be producing similar anti-predatory chemicals that could also benefit its cohabitants.

Despite the passage of over one hundred years since the discovery of the *M. bouillonii*—*A. frontalis* association, there have been strikingly few ecological investigations of either species. The weaving behavior of *A. frontalis* was first described in 1913 (Cowles 1913). This was built upon in a 1966 report that briefly described the habitat of *M. bouillonii* and *A. frontalis* colonies and detailed how pairs of shrimp interact with *M. bouillonii* in the laboratory to construct shelters (Fishelson 1966). Cruz-Rivera and Paul (2002, 2006) further described the commensal nature of this association in their studies of cyanobacteria as sources of food and shelter on coral reefs, highlighting the specificity of the *M. bouillonii*—*A. frontalis* pairing, articulating the protective behaviors observable in the shrimp, and noting the shrimp’s use of the cyanobacteria for both food and shelter. Various other manuscripts also noted these interactions, but the nature of their association and the extensive structures created through the remarkable weaving skills of these shrimp remain to be elucidated.

Without detailed ecological studies, it is difficult to characterize *M. bouillonii* and *A. frontalis* as an emerging threat to coral reef health and resiliency. Experiments measuring effects of the cyanobacteria coming into contact with corals suggest that *M. bouillonii* natural products are toxic to corals and/or their symbionts and may serve as allelopathic agents that provide a competitive advantage against healthy corals (Titlyanov, Yakovleva and Titlyanov 2007). Additionally, localized blooms of *M. bouillonii* near Okinawa were reported as a major driver of mortality for the gorgonian *Annella reticulata* (Yamashiro, Isomura and Sakai 2014). Although somewhat in contrast to the historical knowledge of this species-pair’s distribution and habits on coral reefs, these studies curiously suggest a complexity and context dependence for the ecological roles played by *M. bouillonii* and *A. frontalis*.

Herein, we describe efforts towards a more thorough and expansive understanding of *M. bouillonii* and its symbiotic association with *A. frontalis* in the context of coral reef systems found within the Mariana Islands. We achieve this by combining historical perspectives along with contemporary field studies and experiments. Moreover, we attempt to clarify the ecological relationships of this symbiotic pair among the stony corals with which they become associated, hypothesizing that the engulfing overgrowth of gorgonians by *M. bouillonii* as reported by Yamashiro et al. (2014) may not represent *M. bouillonii* ecology within all reef ecosystems. We address this hypothesis through in situ observations and photography, quantitative surveys of abundance and habitat, and culturing experiments designed to measure how *M. bouillonii* may benefit from its association with *A. frontalis*. With this foundation of knowledge, we hope to expand knowledge regarding the ecology of *M. bouillonii* and *A. frontalis* on coral reefs.

## Methods

### Surveys and Underwater Photography

**Fig. 2:**
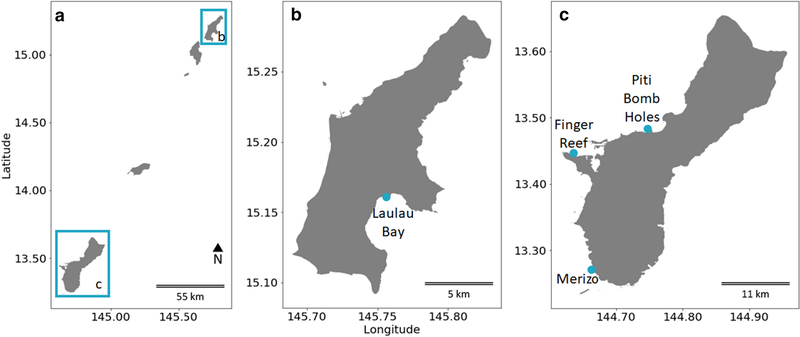
Maps of **a** The Mariana Islands, **b** Saipan, and **c** Guam with marked survey sites. *M. bouillonii*—*A. frontalis* colony growth form and abundance was surveyed on the reefs in Laulau Bay, Saipan, and Finger Reef, Apra Harbor, Guam, while colony substrate preference was surveyed on Finger Reef, on the reef at Piti Bomb Holes, Guam, and on a fringing reef in Merizo, Guam. Sites are marked on the maps with blue dots. The map was created using a NOAA-produced coastline shapefile (NOAA 2005)

Abundance surveys were conducted in the Mariana Islands (Fig. 2a) at Laulau Bay, Saipan, Commonwealth of the Northern Mariana Islands, USA (15°09′18“N:145° 42′20”E; June 17, 2017) (Fig. 2b), and Finger Reef, Apra Harbor, Guam, USA (13° 26′40“N:144° 38′11”E; July 1, 2017) (Fig. 2c). Each consisted of two 50-m transects, haphazardly placed in areas of high *M. bouillonii* abundance. All *M. bouillonii* colonies within 1 m of either side of the transect that displayed morphologies consistent with shrimp association (e.g., displayed the “cobweb,” woven, tube-forming morphology indicative of recent weaving activity by shrimp.) were recorded (100 m^2^ per transect). Shrimp were not often visible during surveys but could reliably be detected by coming into gentle contact with surfaces of *M. bouillonii* that displayed shrimp-associated morphologies and waiting for the distinct, and often startling “snap” of a defending shrimp. Colonies were also photographed. *M. bouillonii* growing without shrimp, which has previously been reported on Guam (Matthew, Schupp, and Luesch 2008), was occasionally observed near transects at Finger Reef growing beneath overhanging reef structures. These cyanobacterial growths were not included in colony counts as the growth morphology is much more dispersed and without discrete, distinct colonies. Records were kept for each consecutive 20-m^2^ area (10 linear meters) in order to capture the local variability and patchiness in colony distribution at these smaller scales. Surveys of each transect were conducted simultaneously by two different SCUBA divers experienced in recognizing *M. bouillonii*—*A. frontalis* colonies, after which the midpoints between the counts of both divers were calculated per 20-m^2^ segment. The Laulau Bay abundance surveys were conducted along the 10- and 12-m depth contours, while the Finger Reef abundance surveys were conducted along the 7- and 12-m depth contours on the reef slope. Means and sample standard deviations for colonies/m^2^ were calculated using each 10-m segment along the 50-m transects as replicates and computed using Microsoft Excel. The purpose of the abundance surveys was to quantitatively characterize the concentration and distribution of *M. bouillonii* colonies across different reefs. They also allowed for the photographic documentation of the varied growth morphologies presented by colonies.

Substrate surveys were conducted at Finger Reef, Apra Harbor, Guam, USA (13° 26′40“N:144°38′11”E; May 31, 2018), Piti Bomb Holes, Guam, USA (13° 28′15“N:144°42′04”E; June 1, 2018), and a fringing reef shoreward of Cocos Lagoon in Merizo, Guam, USA (13° 16′12“N:144°39′42”E; June 13, 2018) (Fig. 2c). Each consisted of two to three 50-m transects, haphazardly placed in areas of high *M. bouillonii*—*A. frontalis* colony abundance. One survey at Finger Reef was conducted on the reef flat, between 1 and 2 m depth, and the other was conducted on the reef slope around the 9-m depth contour. Surveys at Piti Bomb Holes were conducted between less than 1 and 2 m on the reef flat near its boundary with the reef slope. The surveys on the Merizo fringing reef were conducted between 1 and 3 m at the boundary between the reef flat and reef slope. All *M. bouillonii* colonies within 1 m of either side of the transect that displayed morphologies consistent with shrimp association were photographed, providing a record of the substrate occupied. Colonies were attributed substrate types based upon the structure they were contacting; in cases where colonies were contacting multiple substrates, substrate was assigned based on the majority of contacts. Videos were also recorded along each transect to document surrounding benthic habitat and provide additional context for determining substrate occupied by colonies with multiple contacts. Both photographs and videos were captured using a Canon PowerShot^®^ ELPH 100 HS set for underwater macro-photography or video mode, respectively. Additional observational photographs were made via SCUBA from various locations on Guam, and via snorkeling in Laulau Bay, Saipan. The purpose of the substrate surveys was to ascertain the frequencies and site specificity of substrate occupation, thereby providing additional information about how *M. bouillonii* colonies grow on Marianas’ reefs.

### Taxonomic identifications

*Moorena bouillonii*—*A. frontalis* colonies were identified in the field based on their distinctive woven-tube morphology and deep red color (Fig. 1a). Moreover, snapping shrimp such as *A. frontalis* defend against other organisms that physically disturb their cyanobacterial colony by rapidly closing their major chela, which results in a distinctive popping noise that announces their presence within the woven structure. In most cases, the visual or audible presence of *A. frontalis* provided further morphotaxonomic verification during our surveys. Care was also taken to examine cryptic locations within the reef structure, as the cyanobacteria-shrimp colonies are often nestled within crevices and around the bases of solid substrata. Small Sects. (1–3 cm^2^) of colonies were collected and examined via compound light microscopy to inspect the woven *M. bouillonii* tubes for other species of cyanobacterial filaments. In addition, partial *16S rRNA* gene sequences were obtained from both shrimp-associated and non-shrimp-associated *M. bouillonii* for final confirmation of field identifications (see Fig. S1 and Supplementary Methods).

### Culturing experiment

*Alpheus frontalis* and *M. bouillonii* growing with (shrimp-associated cyanobacterium) and without *A. frontalis* (non-shrimp-associated cyanobacterium) were collected from Apra Harbor, Guam (with additional shrimp collected near Merizo, Guam) and used to assemble four different growth conditions in reusable plastic food storage containers: shrimp-associated cyanobacterium with a shrimp, shrimp-associated cyanobacterium without a shrimp, non-shrimp-associated cyanobacterium with a shrimp, and non-shrimp-associated cyanobacterium without a shrimp, each with five replicates. Weighted containers were covered with window screen, placed in a blocked arrangement, and submerged in a flow-through raw seawater table. Submersion allowed for water exchange and the deposition of materials for shrimp to scavenge in each container, while the mesh was intended to prevent shrimp escape. The water table was covered with 50% shade cloth to limit light stress. The experiment was conducted over 15 days (June 11, 2016, to June 26, 2016), with photographs taken each day to document changes in *M. bouillonii* pigmentation and wet weights of cyanobacterial biomass recorded on the first and last days. Excess water was removed from cyanobacterial biomass prior to wet weight measurements using a salad spinner. For each sample, the salad spinner was pumped 20 times at a rate of 1 pump per second, followed by a 10-s period of unconstrained spinning. Statistical analysis of changes in wet weight were conducted using the statsmodel package for Python 3 to build a two-way ANOVA model. Type of cyanobacteria (shrimp versus non-shrimp) and presence or absence of shrimp were set as independent variables for the two-way ANOVA, and an additional term was added to account for the blocked experimental design.

### Nutrient analyses

Five paired water samples were collected from Finger Reef, Apra Harbor, Guam on July 3, 2017. Syringes (50 mL) with blunt-tip needles were used to collect water from inside the woven tubes and chambers of five individual shrimp-associated *M. bouillonii* colonies, and from approximately one meter above each colony in the water column. Samples were flash frozen in dry ice and kept frozen until they were submitted for nutrient analyses at the Oceanographic Data Facility, Scripps Institution of Oceanography, San Diego, California. Statistical significance was determined for each nutrient using Student’s t-test (paired, two-tailed), administered in Microsoft Excel.

## Results

### Abundance surveys

**Fig. 3:**
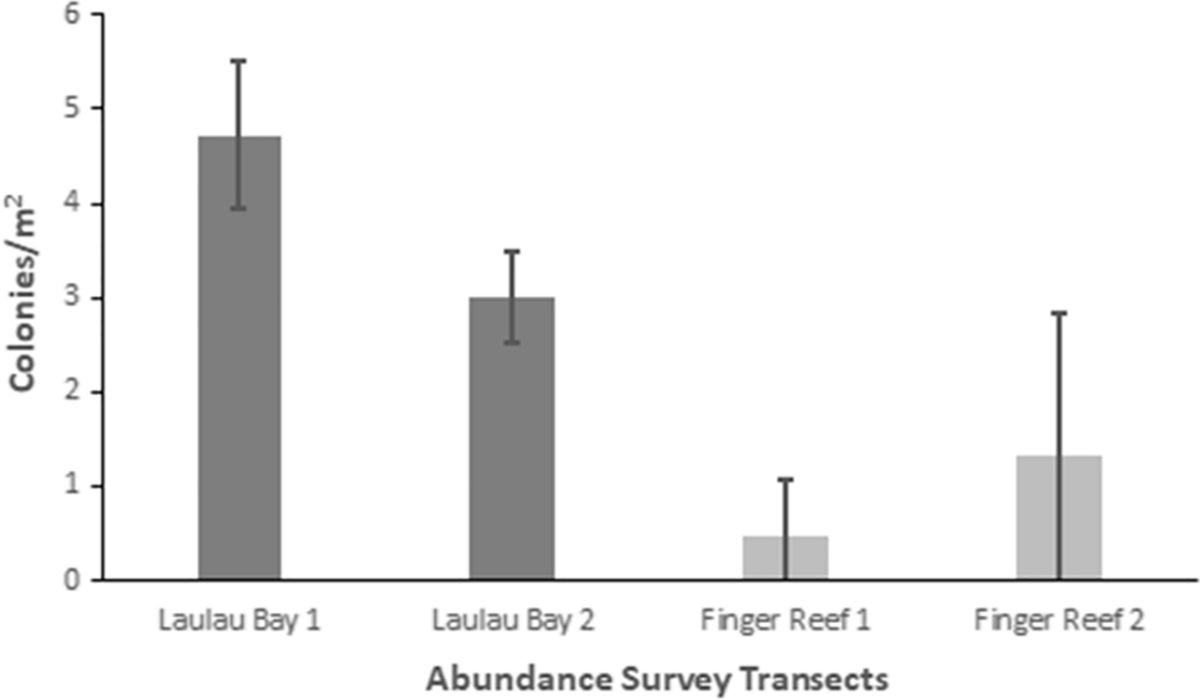
*M. bouillonii*—*A. frontalis* colony density and abundance. *M. bouillonii*—*A. frontalis* colonies were found to be consistently abundant and densely occupying the reef in Laulau Bay, while colony density and abundance was more varied on Finger Reef. Note: For each transect, colonies along each linear 10 m (20 m^2^) of the 50-m transect were separately counted and recorded and make up the replicates represented by the standard deviation error bars

Field abundance surveys of *M. bouillonii*—*A. frontalis* colony growth in Laulau Bay, Saipan (Fig. 2b) and at Finger Reef in Apra Harbor, Guam (Fig. 2c), indicate that colony density varies greatly within and among reefs. The two transects on the reef in Laulau Bay revealed higher densities of *M. bouillonii*—*A. frontalis* colonies (mean = 4.73, 3.01 colonies per m^2^; standard deviation (sd) = 0.79, 0.49; *n* = 5) than the two transects on the reef slope of Finger Reef (mean = 0.47, 1.33 colonies per m^2^; sd = 0.61, 1.50; *n* = 5) (see Fig. 3 and Supplementary Table S1). In consideration of colony density at a smaller scale, it is of note that the most densely populated transect surveyed in Laulau Bay was composed of the top five most densely populated 10 m increments (5.8, 5.2, 4.5, 4.2, and 3.9 colonies per m^2^); however, the sixth most densely populated 10-m segment of reef was surveyed on Finger Reef, with 3.8 colonies per m^2^. Every 10-m interval along the Saipan transects recorded densities greater than two *M. bouillonii*—*A. frontalis* colonies per square meter, and the most densely populated Saipan transect in particular represents an area of consistent high-density *M. bouillonii*—*A. frontalis* colony growth (mean = 4.73 colonies per m^2^; sd = 0.79; *n* = 5). The two transects in Guam, however, ranged from 3.75 to 0.05 colonies per m^2^ and from 1.5 to 0.08 colonies per m^2^, with the former illustrating wide variation in colony density even within small spatial scales (sd = 1.50). Of particular note, while many colonies were observed to be in contact with stony coral, none of the colonies observed during the abundance surveys were found to display morphologies that would suggest coral overgrowth (e.g., cyanobacterial biomass engulfing the extended, external surfaces of corals)(see Supplementary Fig. S2).

### Substrate surveys

**Fig. 4:**
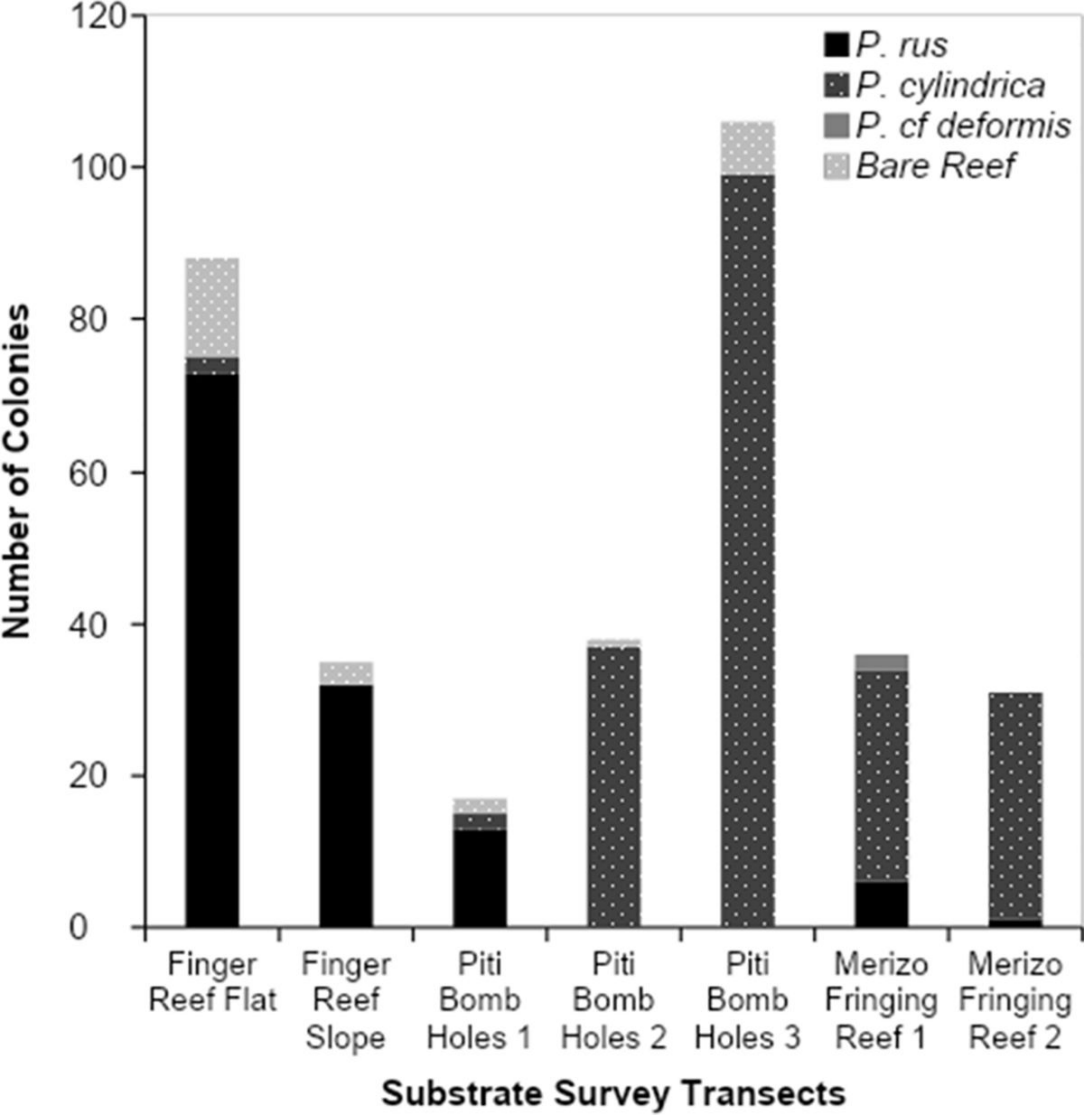
*M. bouillonii*—*A. frontalis* colony substrate distribution. *M. bouillonii*—*A. frontalis* colonies were most often found occupying interstitial spaces among *Porites rus* and *P. cylindrica* corals and were not found to display growth morphologies indicative of coral overgrowth

Substrate surveys conducted around the island of Guam at Finger Reef in Apra Harbor, Piti Bomb Holes, and a fringing reef by Merizo (Fig. 2c) yielded detection of shrimp-associated *M. bouillonii colonies* growing mainly in coral-associated interstitial spaces. Colonies were documented to predominantly grow in association with *Porites rus* and *Porites cylindrica*, with a much smaller number of colonies growing on bare reef substrate and only two colonies found growing with *Porites* cf. *deformis* (see Fig. 4 and Supplementary Table S2). Colonies were not found growing on any other substrate types during these surveys. *Porites rus* was the predominant colony substrate for both the reef flat and reef slope transects at Finger Reef, hosting 83% and 91% of documented colonies, respectively. *P. cylindrica* hosted most of the documented colonies at the Merizo reef, with 78% and 97%, respectively. Transects at Piti Bomb Holes were less consistent, with 76% of the colonies along the first transect growing with *P. rus*, while 97% of colonies along transect two and 93% along transect three were growing with *P. cylindrica*. In agreement with the abundance surveys, while colonies were found to be growing among live coral substrate, none of the colonies observed during the substrate surveys displayed a morphology consistent with coral overgrowth, and macroscopically, none of the corals appeared to be injured by their associated cyanobacteria-shrimp colonies.

### Culturing experiment

**Fig. 5:**
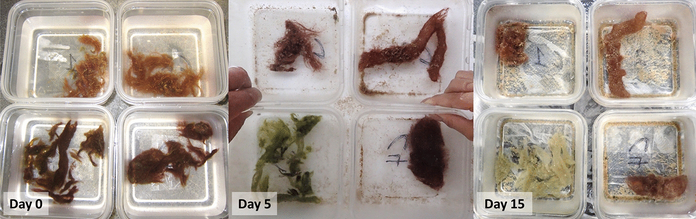
Days 0, 5, and 15 of a representative block (block C) in the photographic time series of the *M. bouillonii* culturing experiment. Representative images from days 0, 5, and 15 of the photo time series of the *M. bouillonii* culturing experiment. Clockwise from top left: non-shrimp associated cyanobacterium without a shrimp, non-shrimp associated cyanobacterium with a shrimp, shrimp associated cyanobacterium with a shrimp, and shrimp associated cyanobacterium without a shrimp

The photographic time series conducted during the culturing experiment revealed generally consistent patterns in pigmentation change for each of the four growth conditions (Fig. 5). Non-shrimp associated cyanobacteria began the experiment with a red-orange hue occasionally accented with yellow-green filaments, which quickly developed into a darker, deeper red hue when grown with shrimp, but remained relatively unchanged when grown without. Shrimp associated cyanobacteria appeared dark red and heavily pigmented at the onset of the experiment. In the absence of shrimp, shrimp associated cyanobacteria dramatically changed appearance, rapidly shifting from dark red filaments to a bright green, gelatinous mass. By day fifteen, the shrimp associated cyanobacteria grown without a shrimp appeared to begin to recover, with new filament growth becoming visible extending out of the green mass. Shrimp associated cyanobacteria grown with shrimp proved to be the least consistent growth condition in this study—two replicates retained dark red pigmentation, two replicates developed patches of green, and one replicate changed to a dark green coloration. This lack of consistency can be explained by the inadvertent removal of the three shrimp living in the three replicates that displayed greening—two died during the course of the experiment (blocks B and D), and one escaped (block A) (see Fig. S3, for complete photographic time course).

**Fig. 6:**
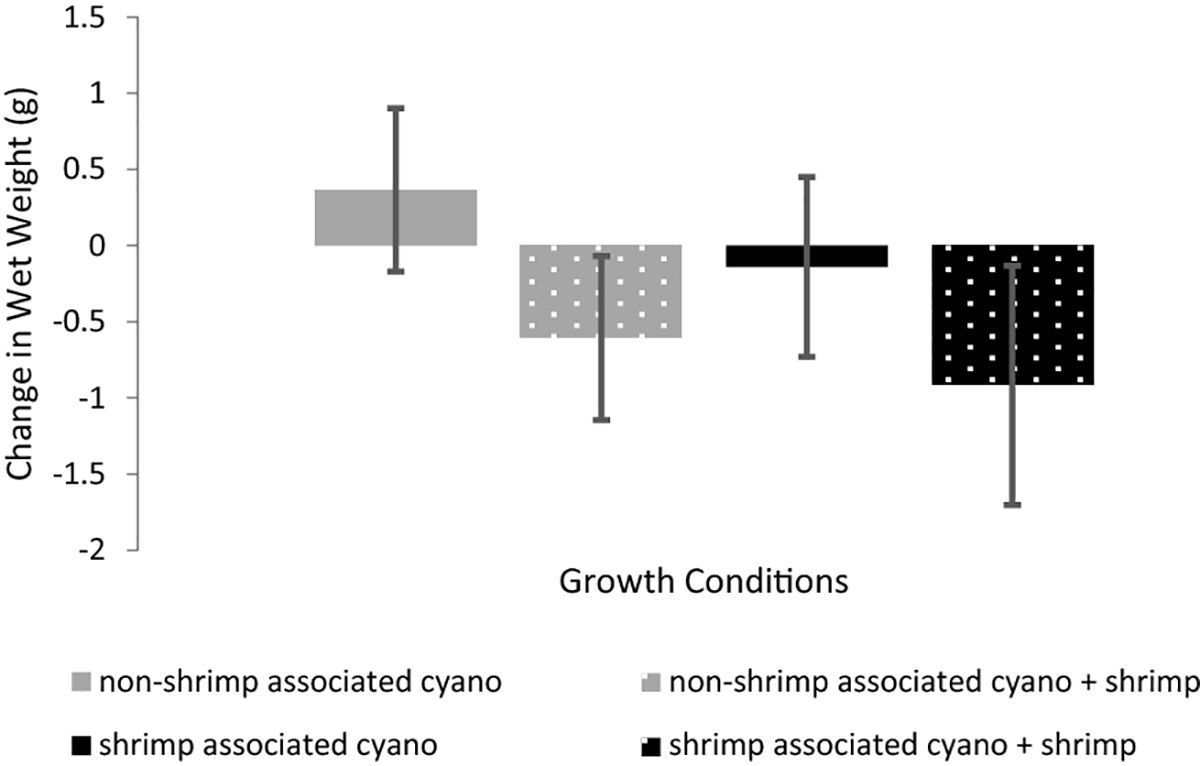
Changes in wet weight from the *M. bouillonii* culturing experiment. Presence of shrimp corresponded to negative changes in wet weight, indicating a loss in cyanobacterial biomass resulting from shrimp feeding. Two-way ANOVA accounting for the blocked experimental design indicated statistically significant effects on changes in wet weight attributable to the type of cyanobacteria (*F* = 7.41, *p* value < 0.05), the presence of shrimp (*F* = 34.14, *p* value < 0.01), and the blocked experimental design (*F* = 10.71, *p* value < 0.01). The interaction between cyanobacterial type and shrimp presence was not found to be significant (*F* = 0.43, *p* value = 0.53)

Out of the four growth conditions, non-shrimp associated cyanobacteria grown without a shrimp was the only condition that averaged net positive growth, as measured by change in wet weight (mean = 0.37 g, sd = 0.54 g, *n* = 5) (Fig. 6, Table S3). Mean change in wet weight was close to zero for shrimp associated cyanobacteria grown without a shrimp (mean = − 0.14 g, sd = 0.59 g, *n* = 5), while both types of cyanobacteria grown with a shrimp decreased in wet weight on average (shrimp associated: mean = − 0.92 g, sd = 0.78 g, *n* = 5; non-shrimp associated: mean = − 0.61 g, sd = 0.54 g, *n* = 5). Two-way ANOVA accounting for the blocked experimental design was administered to test the statistical significance of the effects that cyanobacterial type and shrimp presence had on changes in wet weight. The blocked experimental design (*F* = 10.71, *p* value < 0.01), type of cyanobacteria (F = 7.41, *p* value < 0.05), and presence of shrimp (*F* = 34.14, *p* value < 0.01) were all found to have statistically significant effects on cyanobacterial growth, as measured by changes in wet weight, while the interaction between cyanobacterial type and shrimp presence was not found to be significant (*F* = 0.43, *p* value = 0.53) (Table S4). The significant effect of the blocked experimental design is attributable to the heterogeneity of flow environments in the water table in which the experiment took place; blocks differed in their proximity to inflow and outflow of seawater in the table.

### Comparative nutrient analyses

From Finger Reef, Apra Harbor, Guam, five pairs of water samples were collected from inside *M. bouillonii*—*A. frontalis* colonies and approximately one meter above each colony. The pairs of samples were analyzed for their concentrations of silicate, PO_4_^3−^, NO_3_^−^, NO_2_^−^, and NH_4_^+^. For all five measured nutrients, statistically significant differences in concentration between colonies and ambient seawater were detected (Table 1, Table S5). Colonies were shown to have significantly higher concentrations of PO_4_^3−^, NO_3_^−^, NO_2_^−^, and NH_4_^+^, while ambient seawater was measured to have higher concentrations of silicate.

## Discussion

**Fig. 7:**
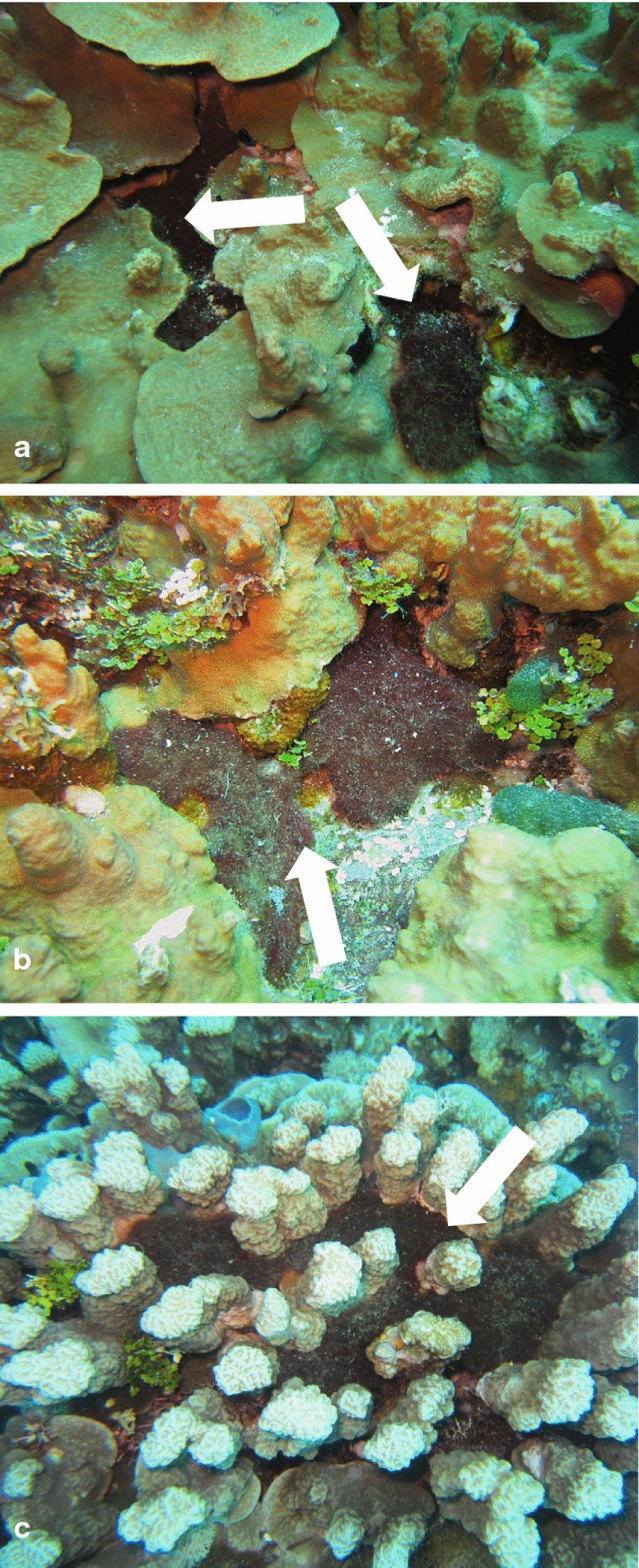
Examples of *M. bouillonii*—*A. frontalis* colony growth habits observed in Saipan and Guam. Colonies of *M. bouillonii* and *A. frontalis* take different growth morphologies related to the substrate they are occupying. Colonies grow **a** Cryptic and recessed in holes and crevices, **b** Entrenched under shelves of hard substrate, and **c** Semi-exposed and winding around columnar structures. Colonies are denoted by white arrows

**Fig. 8:**
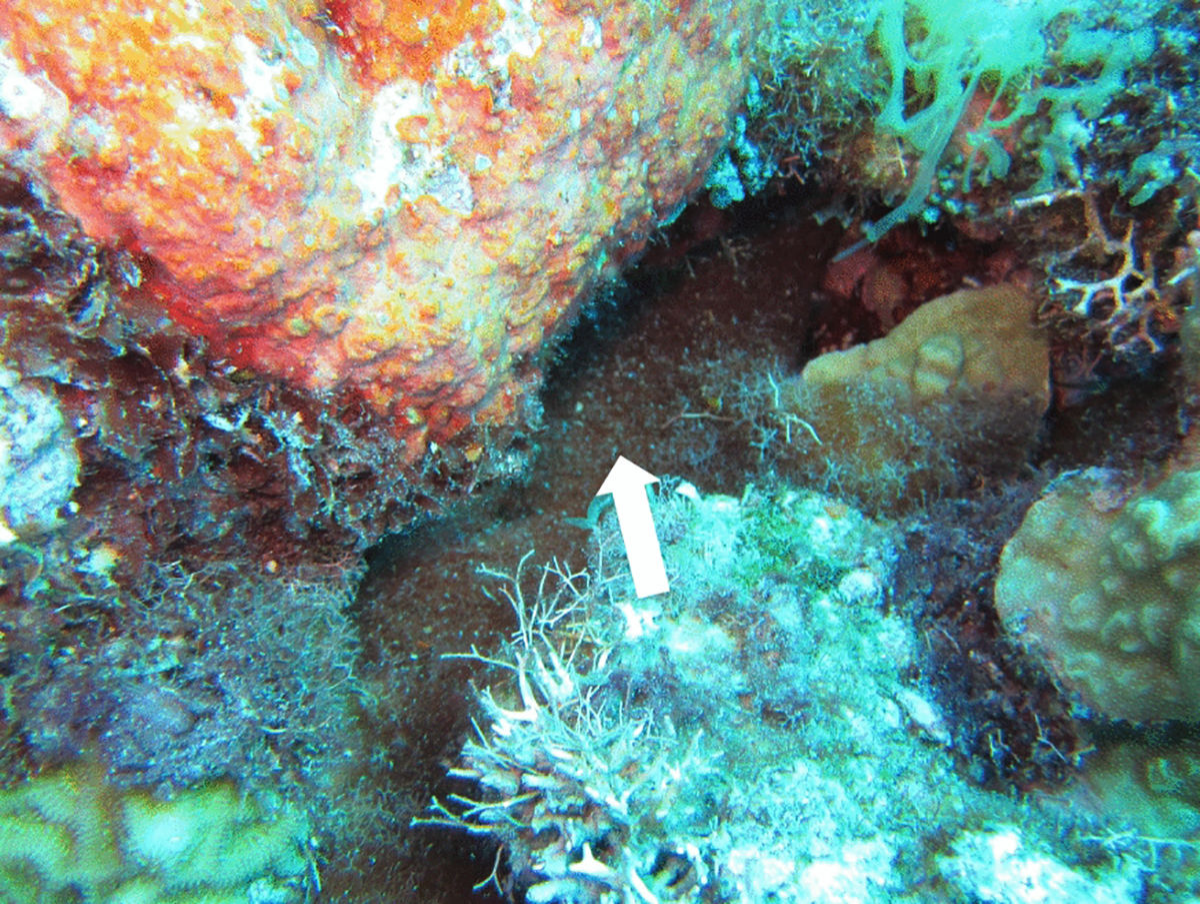
*M. bouillonii*—*A. frontalis* colony growing in Laulau Bay reef crevice. Amidst other algae and cyanobacteria that are overgrowing parts of the reef in Laulau Bay, *M. bouillonii*—*A. frontalis* colonies can be found growing in an apparently benign fashion in interstitial and cryptic spaces. Colony is denoted by white arrow

Study of *M. bouillonii*—*A. frontalis* colonies across the surveyed reefs in Laulau Bay and Apra Harbor allowed for the identification of three common habits of *M. bouillonii* growth (Fig. 7). The first growth form is cryptic and recessed, with colonies growing in crevices and extending deep into or under live or dead coral. The second is entrenched, where colonies extend from under horizontal shelves of coral. The third consists of semi-exposed tubes and chambers of *M. bouillonii* winding between and around upright coral structures, particularly the columnar growths of *P. rus* and the branches of *P. cylindrica*. It is common for large colonies to display multiple growth forms, which appear to be largely driven by the type of benthic structures available for the secure attachment of *M. bouillonii*, and so are highly plastic. On Finger Reef, *P. rus* is extremely prevalent and serves as the major substrate for *M. bouillonii*—*A. frontalis* colonies in this area. Both the entrenched growth form, found extending from under *P. rus* plates, as well as tubes winding around the bases of columnar *P. rus*, were common. In contrast, Laulau Bay is much more varied in its benthic structure, with apparent higher coral species diversity, areas of substantial algal and cyanobacterial growth, and regions of hard substrate devoid of live corals. The cryptic growth form of *M. bouillonii* was particularly common at this site; shrimp-constructed colonies were frequently located in crevices, holes, and other areas not occupied by corals or other algae. Of the close to one thousand colonies that were counted during the abundance surveys and over three hundred fifty colonies documented in the substrate surveys combined over these various reefs, although many colonies were observed in direct contact with corals, none were observed with growth morphologies suggesting overgrowth of these substrates encountered. This suggests that *M. bouillonii*—*A. frontalis* colonies most likely do not interact with stony corals in the same manner as previously reported for the octocoral *A. reticulata* near Okinawa (Yamashiro, Isomura and Sakai 2014). This was particularly notable in Laulau Bay, where there is substantial overgrowth on the reef by other species of cyanobacteria and macroalgae (Fig. 8).

The three *M. bouillonii*—*A. frontalis* colony growth forms observed in this study share the common characteristic of occupying interstitial spaces within and around their substrates, rather than growing over outer exposed surfaces. As the most colonized substrates in our study, the stony corals *P. rus* and *P. cylindrica* are morphologically endowed with a high degree of such interstitial features. *P. rus* grows in a plate form, under which *M. bouillonii*—*A. frontalis* colonies are commonly entrenched, as well as a columnar form, around the bases of which tubes of woven *M. bouillonii* typically wind. Similarly, the branches of *P. cylindrica* provide structures around which tubes of shrimp-associated *M. bouillonii* commonly encircle. The columns of *P.* cf. *deformis*, as well as eroded crevices and columns in old reef structures, provide additional, less frequently inhabited refugia for these cyanobacterial-shrimp colonies.

Two notable substrates that were not documented as being occupied by *M. bouillonii*—*A. frontalis* colonies were soft corals and macroalgae. Along with *P. rus* and *P. cylindrica*, soft coral species of the genus *Sinularia* have been documented to be prevalent at Piti Bomb Holes (Gochfeld 2010), and these soft corals were noted along the transects at this site. Even though *Sinularia* species have branching structures providing substantial interstices, *M. bouillonii*—*A. frontalis* colonies were not found growing among them. In terms of macroalgae, along transects on the Finger Reef slope, tufts of *M. bouillonii* growing independently of shrimp were observed interspersed with *Halimeda* sp. on the underside of overhanging reef structures. The lack of *M. bouillonii—A. frontalis* colonies growing in this fashion might hint at the process by which these colonies are initiated. Shrimp may be collecting free filaments from the underside of overhangs and then moving the filaments to interstitial spaces to begin weaving, resulting in colonies tending to be found in the cryptic benthic spaces known to be favored by snapping shrimp (Johnson, Everest and Young 1947). However, more studies are needed to understand the origins of *M. bouillonii*—*A. frontalis* colonies.

When observations, such as the anecdotal data compiled in the supplemental knowledge aggregation questionnaire (see Supplemental Methods and Results), are combined with the gathered field data, a picture of *M. bouiilonii*—*A. frontalis* colony growth habits emerges that is strikingly similar to that described in historical studies. Both Cowles (1913) and Fishelson (1966) described this species pair as being found under coral rubble and stones, while Hoffmann and Demoulin (1991) assert that *M. bouillonii* habitually grows in the gaps between coral structures and in cavities in the reef, highlighting the cryptic nature of *M. bouillonii*—*A. frontalis* colony growth, and the tendency of these colonies to limit exposure by occupying interstices. Others have described shrimp-associated *M. bouillonii* growing as mats (Engene, Coates and Gerwick 2010; Engene et al. 2012) and tubes (Tidgewell et al 2010) that were attached to debris such as rocks and wood, or growing between coral structures (Matthew, Schupp and Luesch 2008; Engene, Coates and Gerwick 2010).

The most notable difference between the sites surveyed in the present study and that which was studied in Aka Jima by Yamashiro et al. (2014) is that no colonies were found to be associated with gorgonians or other soft corals in Guam or Saipan, whereas colonies in Aka Jima were reported to display an overgrowth morphology exclusively on the surfaces of gorgonians, despite scleractinian coral substrates being available (Yamashiro, Isomura and Sakai 2014). All colonies of *M. bouillonii* and *A. frontalis* encountered thus far within the Mariana Islands displayed variable, substrate-dependent growth forms that favor occupation of interstitial spaces on coral reefs. This suggests that *M. bouillonii*—*A. frontalis* colonies may not exhibit a similar propensity to overgrow habitats dominated by hard coral as to that reported for habitats dominated by octocoral (Yamashiro, Isomura and Sakai 2014). While neither the current study nor the Yamashiro et al. (2014) work directly measured growth dynamics, the static growth morphologies documented between these studies contrast sharply.

Although unusual, evidence of gorgonian tissue damage, and apparent boring and anchoring of cyanobacterial filaments into gorgonian tissue at Aka Jima strongly suggest that the shrimp could sustain *M. bouillonii*—*A. frontalis* colonies long enough to overgrow *A. reticulata*. What are not clear are the factors that may have influenced relatively cryptic, interstitial colonies to transition into conspicuous colonies inhabiting sea fans in Aka Jima. One hypothetical explanation could be that colonies at Aka Jima were initiated by a mechanism alternative to *A. frontalis* weaving *M. bouillonii* directly on the sea fans, such as a typhoon or other dramatic hydrographic activity that could dislodge colonies from the benthos. Such colonies could then have become entangled in the finely branched arms of gorgonians, and subsequently sustained by a shrimp which survived the transplant. Consistent with this hypothesis, Aka Jima has been described in the literature and field station records as having a high frequency of typhoons and other strong oceanographic conditions (Iwao 2018). Dislodgement of benthic cyanobacteria from their growth substrate by wave action has been previously reported in the scientific literature (Becerro, Bonito and Paul 2006). Yamashiro et al. (2014) report that other algal species were also tangled among the gorgonians, and gorgonians are well known to position their growth axis perpendicular to water flow in order to facilitate filtration of food particles from the water column (Leversee 1976). Thus, it is conceivable that dislodged benthic cyanobacterial colonies could become entangled in the finely branched structure of a gorgonian coral.

Furthermore, the observed patterns in pigmentation change, considered alongside the nutrient analyses, help to better characterize the commensal interactions occurring between *M. bouillonii* and *A. frontalis*, and provide further evidence for the notions presented by Yamashiro et al. (2014) in that with *A. frontalis*, the chemically defended *M. bouillonii* could persist on sea fans in nutrient-depleted waters. *M. bouillonii* is potentially gaining, through its association with *A. frontalis*, the benefit of greater access to nutrients via shrimp excrement. By the end of the experiment, the pigmentation of the non-shrimp associated cyanobacteria being grown with a shrimp matched that of the shrimp associated cyanobacteria being grown with a shrimp, suggesting that non-shrimp associated cyanobacteria with exposure to shrimp had access to more nutrients, and so were able to further develop their nitrogen-rich pigmentation (e.g., chlorophyll a and phycoerythrin). In contrast, shrimp associated cyanobacteria that were denied access to shrimp were negatively impacted by their new nutrient-poor environment and required approximately two weeks to acclimate to the new conditions. This interpretation is further supported by the three replicates of shrimp associated cyanobacteria growing with shrimp whose shrimp-driven nutrient influence was removed during the course of the experiment via death or escape; in all three of these cases, cyanobacterial pigmentation deteriorated as the nutrient-supplying shrimp was no longer actively excreting among the filaments. Further support can be found in the results of the nutrient analyses, which found statistically significantly higher levels of phosphate, nitrate, nitrite, and ammonia within the tubes of *M. bouillonii*—*A. frontalis* colonies, as compared to ambient seawater. This suggests that enclosed tubes and chambers of *M. bouillonii* limit water exchange, allowing for shrimp excrements to accumulate and create a beneficial nutrient-enriched environment within their confines.

*A. frontalis* has been described as an obligate tube dweller that benefits from its association with *M. bouillonii* by deriving chemically defended shelter (Fishelson 1966; Banner and Banner 1982; Cruz-Rivera and Paul 2002, 2006). This benefit is exemplified during the collection of *A. frontalis*, as even very short periods of time during which shrimp are not completely enshrouded in cyanobacterial filaments can result in swift predation by invertivorous fishes (Leber, Biggs and Gerwick, unpublished observations). Another reported benefit is use of *M. bouillonii* by *A. frontalis* as a food source; Fishelson (1966) reported observing *A. frontalis* eating filaments of *M. bouillonii* and detecting cyanobacterial cells in shrimp excrement. The changes in wet weight recorded in the culturing experiment, namely the statistically significant effect of shrimp presence on changes in wet weight, provide further support that one benefit shrimp are gleaning from their cyanobacterial home is a source of food. Cultures that included shrimp saw notable decreases in wet weight, resulting from shrimp feeding upon the filaments. This brings into focus a seemingly paradoxical situation where *M. bouillonii* appears to benefit from the nutrient-enrichment provided by *A. frontalis* that allows for the development of its rich pigmentation, while also being limited to a degree in its ability to increase in biomass as *A. frontalis* benefits from *M. bouillonii* as a food source.

While there is more to be learned about the association between *M. bouillonii* and *A. frontalis*, this report expands on the limited knowledge of the habitat ecology of this unique symbiosis through the consideration of patterns in growth morphology in the Mariana Islands. Specifically, we demonstrate that *M. bouillonii* and *A. frontalis* consistently occupy the interstitial spaces of coral structures and other hard substrates, rather than overgrowing the external surfaces of living coral colonies. Furthermore, this report gives more dimension to the commensal partnership between *M. bouilloni* and *A. frontalis* by providing additional evidence to explain how both organisms benefit from their intimate association with each other.

## Supplementary Material

1696932_Sup1

1696932_mov

## Figures and Tables

**Table 1:** Summary of paired water sample nutrient analysis data, revealing statistically significant differences in nutrient concentration between the water column and *M. bouillonii* colonies for all nutrients measured. (*n* = 5)

	NO_3_^−^ μmol/L	PO_4_^3−^ μmol/L	Silicate μmol/L	NO_2_^−^ μmol/L	NH_4_^+^ μmol/L
Water column mean	0.402	0.038	2.3	0.004	0.444
Water column sd	0.165	0.013	0.158	0.009	0.088
Colony mean	1.032	0.148	2.1	0.05	0.678
Colony sd	0.466	0.055	0.187	0.014	0.241
*p* value^a^	0.0200	0.0056	0.0217	0.0016	0.0404

a *p* values from Student’s *t* test (paired, two tailed)

## Data Availability

16S rRNA sequence data associated with this project can be accessed via NCBI under the accession numbers MT826199.1, MT826200.1, MK299234.1, MK299235.1, MT826201.1, MT826202.1, MK299236.1, MK299237.1, MT826203.1, MT826204.1, MT826205.1, MT826206.1, MT826207.1, and MT826208.1. All other data pertinent to this study have been included in this manuscript or in the supplementary information.
