# Supplementary material for: Cyanobacteria-shrimp colonies in the Mariana Islands
Source: Aquat Ecol. 2021 Feb 27;55(2):453–65. doi: 10.1007/s10452-021-09837-6 (PMC8223766; doi:10.1007/s10452-021-09837-6)
Supplement: Supplementary file 1 — Supplementary material 1 (DOCX 4679 kb) [file 10452_2021_9837_MOESM1_ESM.docx]

**Cyanobacteria-Shrimp Colonies in the Mariana Islands**

**Supplemental Information**

Christopher A. Leber^1^, Andres Joshua Reyes^2^, Jason S. Biggs^2^* and William H. Gerwick^1,3^*

**^1^**Center for Marine Biotechnology and Biomedicine, Scripps Institution of Oceanography, University of California, San Diego, La Jolla, CA 92093, USA

^2^University of Guam Marine Laboratory, UOG Station, Mangilao, GU 96923, USA

**^3^**Skaggs School of Pharmacy and Pharmaceutical Sciences, University of California, San Diego, La Jolla, CA 92093, USA

*Corresponding Authors

Please direct correspondence to [biggs.js@gmail.com](mailto:biggs.js@gmail.com) and [wgerwick@ucsd.edu](mailto:wgerwick@ucsd.edu)

**Supplementary Fig. S1** – Phylogenetic tree of shrimp associated and non-shrimp associated *M. bouillonii* partial 16S rRNA gene sequences from Apra Harbor

**Supplementary Fig. S2** *–* Representative photos of *M. bouillonii*-*A. frontalis* colonies on reefs in Saipan and Guam

**Supplementary Fig. S3** *–* Photographic time series of *M. bouillonii* colony growth experiment

**Supplementary Table S1** – *M. bouillonii* – *A. frontalis* colony density and abundance

**Supplementary Table S2** **-** *M. bouillonii* – *A. frontalis* colony substrate distribution

**Supplementary Table S3** – *M. bouillonii* colony growth experiment: changes in wet weight

**Supplementary Table S4** – *M. bouillonii* colony growth experiment: Two-way ANOVA outputs

**Supplementary Table S5** - *M. bouillonii*-*A. frontalis* water column and colony comparative water analyses nutrient data

**Supplementary Table S6** – *M. bouillonii* knowledge aggregation questionnaire questions and responses

**Supplementary Methods** – 16S rRNA gene sequencing

**Supplementary Methods** – Knowledge Aggregation Questionnaire

**Supplementary Results** – Knowledge Aggregation Questionnaire

**Supplementary Video S1** – *A. frontalis* weaving filaments of *M. bouillonii* into structures

**Supplementary Fig. S1** - Phylogenetic tree of shrimp associated and non-shrimp associated *M. bouillonii* partial 16S rRNA gene sequences from Apra Harbor

**
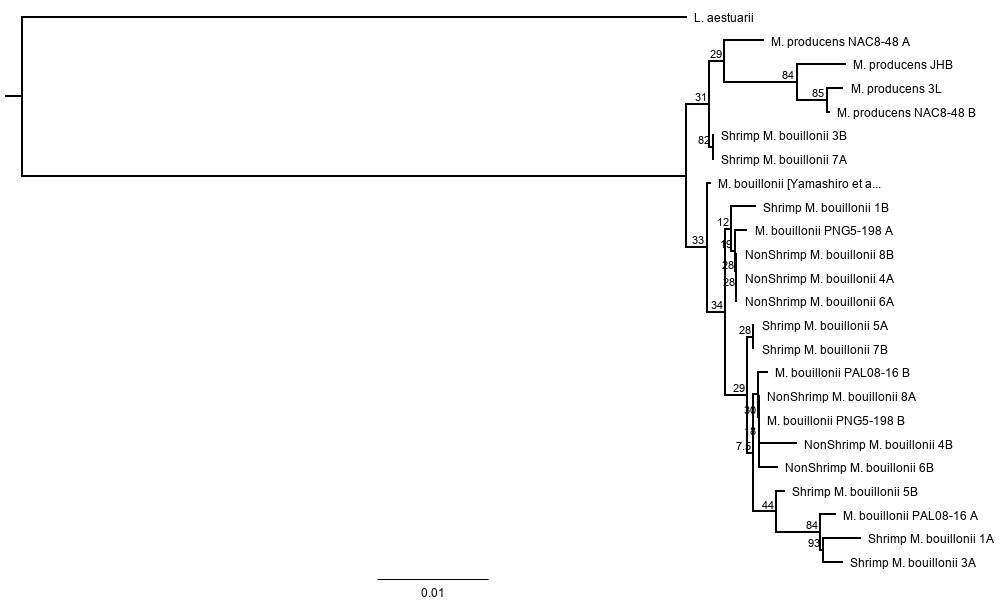
**

**Supplementary Fig. S2** - Representative photos of *M. bouillonii*-*A. frontalis* colonies on reefs in Saipan and Guam

**
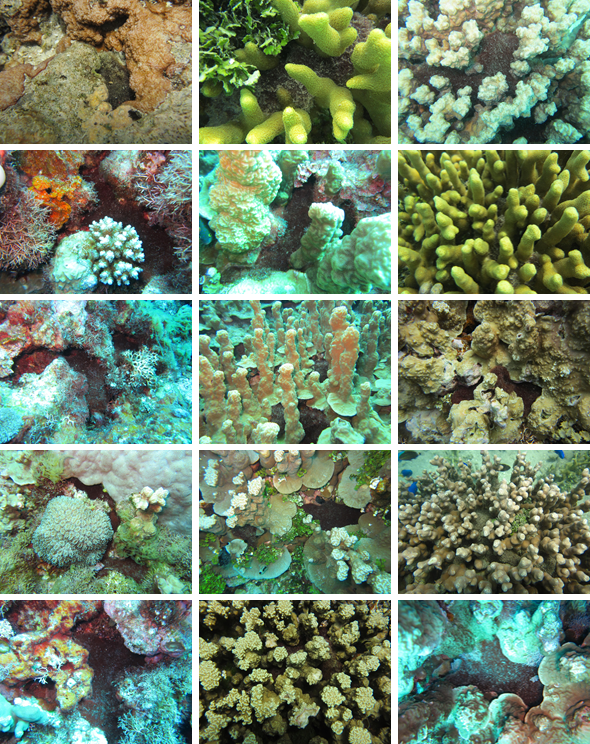
**

**Supplementary Fig. S3** - Photographic time series of *M. bouillonii* colony growth experiment


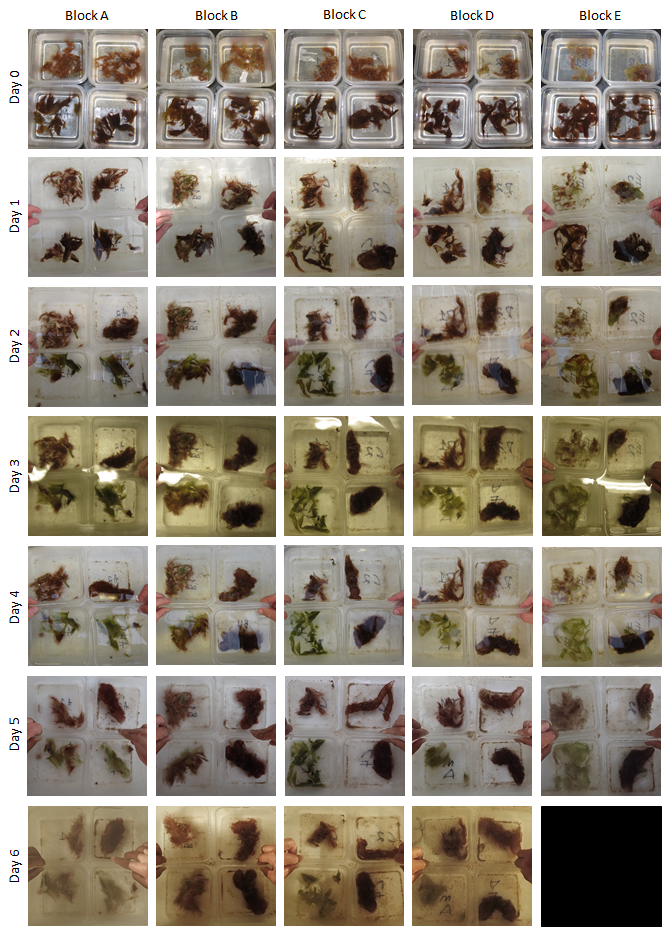


**Supplementary Fig. S3 (cont.)**

**
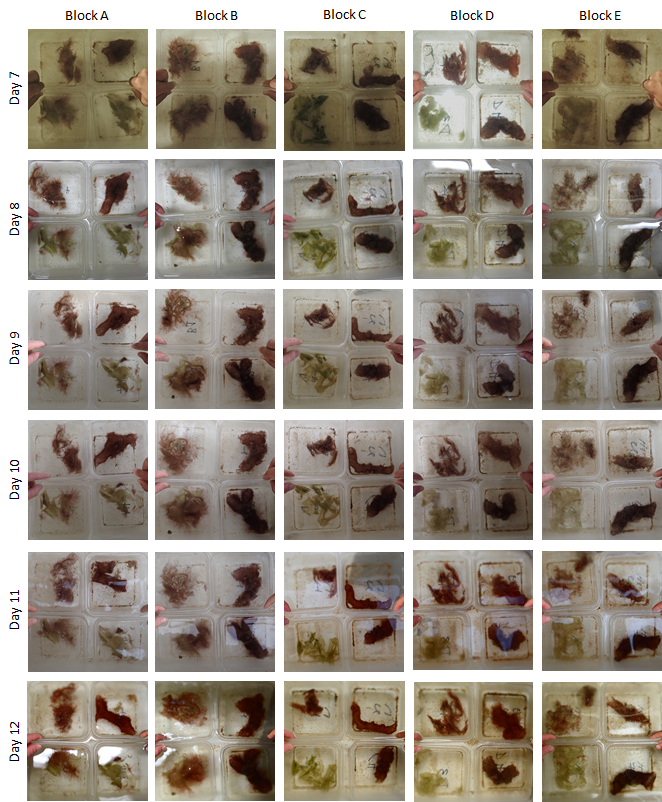
**

**Supplementary Fig. S3 (cont.)**

**
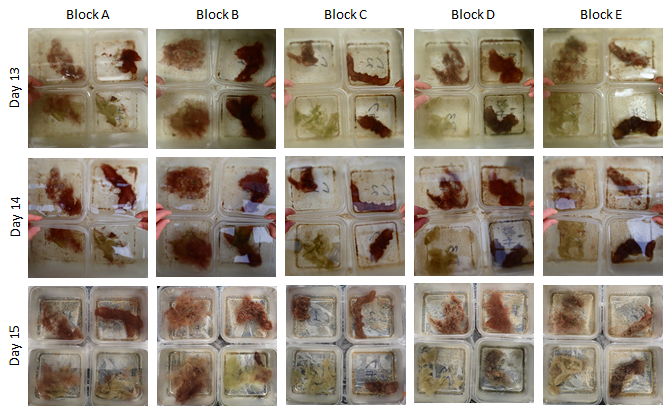
**

Clockwise from top left: non-shrimp associated cyanobacterium without a shrimp, non-shrimp associated cyanobacterium with a shrimp, shrimp associated cyanobacterium with a shrimp, and shrimp associated cyanobacterium without a shrimp.

**Supplementary Table S1** - *M. bouillonii* – *A. frontalis* colony density and abundance

|  | Laulau Bay 1 | | Laulau Bay 2 | | Finger Reef 1 | | Finger Reef 2 | |
| --- | --- | --- | --- | --- | --- | --- | --- | --- |
|  | Colonies | Colonies/m^2^ | Colonies | Colonies/m^2^ | Colonies | Colonies/m^2^ | Colonies | Colonies/m^2^ |
| 0-10 m | 84 | 4.20 | 67 | 3.35 | 30 | 1.50 | 75 | 3.75 |
| 10-20 m | 90.5 | 4.53 | 70 | 3.50 | 10.5 | 0.53 | 32.5 | 1.63 |
| 20-30 m | 116.5 | 5.83 | 57 | 2.85 | 2.5 | 0.13 | 20 | 1.00 |
| 30-40 m | 77.5 | 3.88 | 45 | 2.25 | 2 | 0.10 | 4 | 0.20 |
| 40-50 m | 104 | 5.20 | 62 | 3.10 | 1.5 | 0.08 | 1 | 0.05 |
| Mean |  | 4.725 |  | 3.01 |  | 0.465 |  | 1.325 |
| s.d. |  | 0.786 |  | 0.492 |  | 0.607 |  | 1.497 |

**Supplementary Table S2 -** *M. bouillonii* – *A. frontalis* colony substrate distribution

| Substrate | | Finger Reef  Flat | Finger Reef Slope | Piti  Bomb Holes 1 | Piti  Bomb Holes 2 | Piti  Bomb Holes 3 | Merizo Fringing Reef 1 | Merizo Fringing Reef 2 |
| --- | --- | --- | --- | --- | --- | --- | --- | --- |
| *P. rus* | Count | 73 | 32 | 13 | 0 | 0 | 6 | 1 |
|  | Proportion | 0.83 | 0.91 | 0.76 | 0.00 | 0.00 | 0.17 | 0.03 |
| *P. cylindrica* | Count | 2 | 0 | 2 | 37 | 99 | 28 | 30 |
|  | Proportion | 0.02 | 0.00 | 0.12 | 0.97 | 0.93 | 0.78 | 0.97 |
| *P.* cf. *deformis* | Count | 0 | 0 | 0 | 0 | 0 | 2 | 0 |
|  | Proportion | 0.00 | 0.00 | 0.00 | 0.00 | 0.00 | 0.06 | 0.00 |
| Bare Reef | Count | 13 | 3 | 2 | 1 | 7 | 0 | 0 |
|  | Proportion | 0.15 | 0.09 | 0.12 | 0.03 | 0.07 | 0.00 | 0.00 |
| Total | | 88 | 35 | 17 | 38 | 106 | 36 | 31 |

**Table S3** - *M. bouillonii* colony growth experiment: changes in wet weight

| Block | Factor: Cyano | Factor: Shrimp | Initial (g) | Final (g) | Difference |
| --- | --- | --- | --- | --- | --- |
| A | NS | NS | 3.11 | 3.86 | 0.75 |
| B | NS | NS | 3.22 | 3.79 | 0.57 |
| C | NS | NS | 3.08 | 2.58 | -0.5 |
| D | NS | NS | 3.31 | 3.52 | 0.21 |
| E | NS | NS | 3.35 | 4.15 | 0.8 |
| A | NS | S | 3.34 | 3.02 | -0.32 |
| B | NS | S | 3.68 | 3.82 | 0.14 |
| C | NS | S | 3.11 | 1.97 | -1.14 |
| D | NS | S | 3.11 | 2.02 | -1.09 |
| E | NS | S | 3.7 | 3.07 | -0.63 |
| A | S | NS | 3.38 | 3.35 | -0.03 |
| B | S | NS | 3.38 | 4.02 | 0.64 |
| C | S | NS | 3.24 | 2.3 | -0.94 |
| D | S | NS | 2.35 | 2.42 | 0.07 |
| E | S | NS | 3.57 | 3.13 | -0.44 |
| A | S | S | 3.28 | 2.31 | -0.97 |
| B | S | S | 3.14 | 3.02 | -0.12 |
| C | S | S | 3.37 | 1.16 | -2.21 |
| D | S | S | 2.64 | 2.02 | -0.62 |
| E | S | S | 3.68 | 3.01 | -0.67 |

**Table S4** - *M. bouillonii* colony growth experiment: Two-way ANOVA outputs

|  | **sum of squares** | **df** | **F** | **P-value** |
| --- | --- | --- | --- | --- |
| **factor - cyano** | 0.83232 | 1.0 | 7.406190 | 0.018559 |
| **factor - shrimp** | 3.83688 | 1.0 | 34.141512 | 0.000079 |
| **block** | 4.81510 | 4.0 | 10.711489 | 0.000624 |
| **interaction - cyano:shrimp** | 0.04802 | 1.0 | 0.427294 | 0.525648 |
| **residual** | 1.34858 | 12.0 | NaN | NaN |

**Table S5** - *M. bouillonii*-*A. frontalis* water column and colony comparative water analyses nutrient data

|  | NO_3_^-^ | PO_4_^3-^ | Silicate | NO_2_^-^ | NH_4_^+^ |
| --- | --- | --- | --- | --- | --- |
|  | µmol/L | µmol/L | µmol/L | µmol/L | µmol/L |
| water column 1 | 0.38 | 0.04 | 2.3 | 0 | 0.55 |
| water column 2 | 0.31 | 0.03 | 2.4 | 0 | 0.43 |
| water column 3 | 0.69 | 0.06 | 2.5 | 0.02 | 0.51 |
| water column 4 | 0.34 | 0.03 | 2.2 | 0 | 0.4 |
| water column 5 | 0.29 | 0.03 | 2.1 | 0 | 0.33 |
| colony 1 | 1.59 | 0.19 | 2.3 | 0.07 | 0.95 |
| colony 2 | 0.71 | 0.14 | 2.2 | 0.04 | 0.7 |
| colony 3 | 1.46 | 0.21 | 2.2 | 0.06 | 0.69 |
| colony 4 | 0.86 | 0.07 | 1.9 | 0.04 | 0.76 |
| colony 5 | 0.54 | 0.13 | 1.9 | 0.04 | 0.29 |
| p-value^a^ | 0.020000 | 0.005571 | 0.021743 | 0.001556 | 0.040447 |

^a^P-values from Student’s T-test (paired, two tailed)

**Table S6** - *M. bouillonii* knowledge aggregation questionnaire questions and responses

| Questions | Answers |
| --- | --- |
| During which years were you involved with 𝘔. 𝘣𝘰𝘶𝘪𝘭𝘭𝘰𝘯𝘪𝘪 collection or field study? | 2011 and 2013 |
|  | 2000 |
|  | 2006, 2009 |
|  | 2000-2015 |
|  | 1993-present |
|  | 2002-2004 |
|  | 2008 to 2012 |
|  | 2014, 2016 |
|  | March 2016 to July 2017 |
|  | 2000 - 2016 |
|  | 2005-2008 |
|  | 2002 - 2009 |
|  | 2012-2016 |
|  | 2000-2004 (collection in 2002) |
|  | 2006 |
|  | 2016 to present |
|  | 2007 & 2009 |
| Where did you collect or study 𝘔. 𝘣𝘰𝘶𝘪𝘭𝘭𝘰𝘯𝘪𝘪? | Puerto Rico and Saipan |
|  | Various reef systems at the northern coast of New Britain, Papua New Guinea. |
|  | Papua New Guinea (near Alotau & Milne Bay); Palmyra Atoll |
|  | Papua New Guinea, Costa Rica, Panama |
|  | Guam |
|  | Papua New Guinea (Bismark Sea) and Oahu Hawaii |
|  | Palmyra Atoll |
|  | American Samoa, Guam |
|  | Apra Harbor, Guam; Mamaon Channel (Merizo), Guam; Piti, Guam |
|  | Papua New Guinea, Palmyra Atoll, Lakadeep Islands, Guam, Saipan |
|  | Panama (i think) |
|  | Papua New Guinea - off the coast of Rabaul and New Ireland |
|  | Panama, Saipan, American Samoa |
|  | Papua New Guinea |
|  | Papua New Guinea |
|  | Guam, Saipan |
|  | Papua New Guinea & Palmyra Atoll |
| How would you rank your ability to accurately identify 𝘔. 𝘣𝘰𝘶𝘪𝘭𝘭𝘰𝘯𝘪𝘪 in the field? (1: no ability, 5: optimal ability) | 3 |
|  | 5 |
|  | 5 |
|  | 5 |
|  | 5 |
|  | 4 |
|  | 2 |
|  | 5 |
|  | 5 |
|  | 5 |
|  | 3 |
|  | 5 |
|  | 5 |
|  | 5 |
|  | 5 |
|  | 5 |
|  | 5 |
| In your own words, please describe 𝘔. 𝘣𝘰𝘶𝘪𝘭𝘭𝘰𝘯𝘪𝘪 colony growth on coral reefs, including any patterns that you observed.  (Responses modified to maintain anonymity) | A dense assemblage with lighter or bleached filaments and a minimal mucous layer near the exterior of the colony and dark brightly colored fresh filaments within the assemblage that were not directly exposed to the elements. |
|  | This particular cyanobacterial species is usually found in shallow warm waters and is readily recognized in the field by its thallus morphology. The thalli form tenacious mats and are found attached to either dead corals of the Acropora species or other calcareous matter. |
|  | Papua New Guinea - A reddish cobweb form in reef crevices (w/snapping shrimp); Palmyra - a reddish tube form in reef crevices. Both sites were ~20-50 ft depth |
|  | Grows in small, flat mat across small openings in the reef, typically at depths between 10-25 meters. Often associated with snapping shrimp. |
|  | The most obvious growth morph of M.b. is cocoon woven through and deep into living and/or dead corals (often Porites rus) by a shrimp. Free living morphs tend to be much more of a "puffball" with strings extending out. Both have a distinctive red to maroon pigmentation to them. |
|  | It has been a long time, but I remember finding areas where it would form thick mats on dead coral. |
|  | Upright tufts |
|  | Usually arranged in shrimp nests. Occasionally free-growing or disorganized. |
|  | First, the "free-growing tufts" (the unknitted algal strings) that we collected at Gab Gab Beach 1(Apra Harbor) had a greenish-red color, whereas the tufts (knitted by A. frontalis) within the coral had a dark red color. Second, the tufts that we collected at Middle shoals (Apra Harbor) fit the same profile. Most of the tufts we collected at this site were a dark red color and within coral, presumably knitted by the shrimp. |
|  | grows as coverings over holes in reef, or as tubes around the base of corals and rocks |
|  | as i recall, long tufts of dark green (in marine environment) filaments on sandy/loose substrate |
|  | Attached to coral, grows in sheets with a cross-hatch pattern. |
|  | The typical growth morphology found is the "woven" form, assembled by the pistol shrimp. This can appear slightly different in each case, but generally colonies will have a few tubes that run through the coral heads and connect larger chambers. These chambers can be somewhat buried into the coral, or in some cases exposed to the water column. Usually, the tubes and chambers are a deep red color and tend to be fairly clean from marine debris. The tunnels can be intricate, and depending on the coral they are growing on, they can be difficult to spot and collect.  Additionally, some tufts of M.b. can be found unwoven, but it is much more rare. These typically look like a typical "hairball" of red filaments, and they tend to contain more discolored filaments. |
|  | It always had a very cobweb-like appearance and was often draped over the opening of homes for snapping shrimp, about the size of a silver dollar. The shrimp would often make popping noises when you would steal their cover. When draped like that the surface was opaque and you could not see through it. The color of the algae was a deep wine-colored red. It was growing on the corals or had been placed there by the shrimp, but it was not extensively growing. Only showed up in pockets or small areas where we were collecting. |
|  | […] |
|  | Grows with (in woven tubes and chambers, winding between coral heads and columns, through crevices in the reef, etc) and without shrimp (loose tufts, typically under overhanging coral structures) |
|  | Moorea boillonii was often found as a patch of red filamentous growth among coral. It is a blood red color and fairly bright when a flash is used. The filaments are often in a somewhat round patch when observed in PNG and found weaving around coral in Palmyra. This pattern was discussed with Bill when it was observed in the field and we thought that it might have to do with the shrimp that is almost always found inside. The growth was never completely covering the coral it was found with. It was always a relatively confined growth to the interior of the coral or bottom. Not on the top. |
| On a scale from 1-5, how would you characterize the degree to which you observed 𝘔. 𝘣𝘰𝘶𝘪𝘭𝘭𝘰𝘯𝘪𝘪 colonies overgrowing corals?  (1: no overgrowth, 5: complete overgrowth) | 2 |
|  | 1 |
|  | 1 |
|  | 3 |
|  | 1 |
|  | 4 |
|  | 3 |
|  | 2 |
|  | 1 |
|  | 2 |
|  | 2 |
|  | 3 |
|  | 1 |
|  | 2 |
|  | 2 |
|  | 1 |
|  | 3 |
| On a scale from 1-5, based on what you observed in the field, how would you characterize the relationship between 𝘔. 𝘣𝘰𝘶𝘪𝘭𝘭𝘰𝘯𝘪𝘪 colonies and coral reefs? (1: harmonious, 5: in conflict) | 2 |
|  | 2 |
|  | 3 |
|  | 1 |
|  | 2 |
|  | 4 |
|  | 3 |
|  | 2 |
|  | 3 |
|  | 2 |
|  | 2 |
|  | 2 |
|  | 1 |
|  | 2 |
|  | 2 |
|  | 1 |
|  | 3 |
| On a scale from 1-5, based on what you observed in the field, how would you characterize the impact of 𝘔. 𝘣𝘰𝘶𝘪𝘭𝘭𝘰𝘯𝘪𝘪 colonies on coral reefs? (1: benign, 5: deleterious) | 2 |
|  | 1 |
|  | 1 |
|  | 3 |
|  | 1 |
|  | 4 |
|  | 3 |
|  | 2 |
|  | 3 |
|  | 1 |
|  | 2 |
|  | 2 |
|  | 1 |
|  | 2 |
|  | 2 |
|  | 1 |
|  | 3 |
| On a scale from 1-5, based on what you observed in the field, how would you characterize the degree to which 𝘔. 𝘣𝘰𝘶𝘪𝘭𝘭𝘰𝘯𝘪𝘪 colony growth on coral reefs is recessed vs exposed? (1: cryptic & recessed, 5: overexposed) | 1 |
|  | 3 |
|  | 2 |
|  | 2 |
|  | 1 |
|  | 3 |
|  | 2 |
|  | 2 |
|  | 3 |
|  | 2 |
|  | 3 |
|  | 3 |
|  | 3 |
|  | 3 |
|  | 5 |
|  | 2 |
|  | 1 |
| Any additional observations on the growth of 𝘔. 𝘣𝘰𝘶𝘪𝘭𝘭𝘰𝘯𝘪𝘪 that were not addressed in the above questions? (Responses modified to maintain anonymity) | In Saipan the largest assemblage was interwoven between a coral. There the assemblage didn't seem to be overgrowing the coral, I make that judgement because i didn't see it protruding out over the surface of the coral but woven under the exterior arms of the coral and because it didn't seem very tightly woven in with the coral, where it could be pulled out rather easily. That colony was also associated with snapping shrimp which snapped at us as we tore up the assemblage. |
|  | Snapping shrimps are found to be associated with field collection of this cyanobacterial species. |
|  | M. bouillonii is probably not obvious to the non-trained eye, but once you know what to look for you see it quite often. In Palmyra we only saw it on the open reef on SCUBA (one of the few cyanos out there), while other filamentous cyanobacteria were more common in the lagoon or lower flow/shallow areas (snorkeling or collecting by hand). I also don't think we saw it snorkeling in shallow areas in PNG, just on SCUBA. |
|  | Growth of the cyanobacterium was opportunistic on the coral reef system, but not harmful and provides habitat for snapping shrimp. |
|  | I have known, worked on, and collected this cyano for decades and have a intimate knowledge of its presence on Guam. Within Apra Harbor, you often see "tubes" of Mb woven through the architectural framework of columnar Porites rus collonies. The shrimp weaves this cocoon into a multi-branched network, and purposefully attaches this to points along the way. Much of the cocoon does not come into direct contact with live coral tissue, and when collected, the once shaded areas of the colony do not appear discolored. In fact, although the places in which these cocoons enter into the interior crevices are often "dead" (i.e., with a distinct colony edge) these entrance areas look the same in other colonies that are devoid of shrimp. This is the same for P.rus plates, as they are often "tucked" far enough back as to be growing where direct sunlight does not reach. Outside of Apra, Mb cocoons are also prevalent on both fore- and back-reef habitats. On fore-reefs, Mb can be most readily found in pockets and holes of the pavement and thus, it also exists quite readily where corals are not growing. |
|  | It often growing as mats on coral sand as well as on dead coral. In an area north of Papua New Guinea I remember finding a lot of M. boullonii in areas that recently had undergone significant coral bleaching. |
|  |  |
|  |  |
|  | none at this time |
|  |  |
|  |  |
|  |  |
|  | Besides the places I collected it, I also saw M.b. in the Philippines (in Mabini, Batangas). I would guess it is pan-tropical, now that I know what to look for I can find it on almost any reef dive I take. |
|  | […] |
|  | […] |
|  |  |
|  | I took many pictures of the colonies and patches that were observed in Palmyra. There were less pictures from PNG. I also happened to observe what I believe was M. bouillonii in Thailand during a vacation in 2013. I was near Ko Pi Pi on a relatively shallow dive near an overhang. The cobweb morphology and the color was obvious! I didn't collect the sample and I did not probe to see if a shrimp was observed. |

**Supplementary Methods – 16S rRNA gene sequencing**

Sections of *M. bouillonii* – *A. frontalis* colonies and non-shrimp associated *M. bouillonii* were collected in bulk from Apra Harbor, Guam on 10 June 2016 and 15 June 2017. RNAlater solution was used to store preserved biomass samples at -20°C until processing. One to two aliquots of biomass from each of four samples (two shrimp associated and two non-shrimp associated) RNAlater sample were blotted dry with paper towel and macerated with mortar and pestle in liquid nitrogen. Frozen homogenized aliquots were processed via the G20 Genomic Tip - Qiagen bacterial DNA isolation protocol, followed by cleanup via G20 genomic tip, by manufacturer’s instructions (Qiagen). Universal specific cyanobacterial primers were used to amplify partial 16S sequences, using 106F (CGGACGGGTGAGTAACGCGTGA) and 781R(a) (GACTACTGGGGTATCTAATCCCATT) (Nübel, Garcia-Pichel and Muyzer 1997). Taq polymerase (Promega) was used, with an extension time of 1:00. Products were inserted into a cloning vector by TOPO-TA cloning kit (Life Technologies) into *E. coli* DH5α as per manufacturer’s instructions. Colonies were generated overnight, transferred to liquid media and then grown overnight again. The QIAprep Miniprep kit and protocol (Qiagen) were used to harvest plasmids, followed by Sanger sequencing with M13 forward and reverse primers of two colonies per original aliquot. Sequences were compared using, and a phylogenetic tree was generated using Geneious (Geneious v2019.2). All sequences, including those featured for reference in the phylogenetic tree, are available via NCBI GenBank at the following accession numbers: *L. aestuarii* = NR_114680.1; *M. producens* NAC8-48 A = GU724199.1; *M. producens* NAC8-48 B = GU724200.1; *M. producens* JHB = FJ151521.1; *M. producens* 3L = FJ151527.1; *M. bouillonii* [Yamashiro et al.] = AB922817.1; *M. bouillonii* PNG5-198 A = FJ041298.1; *M. bouillonii* PNG5-198 B = FJ041299.1; *M. bouillonii* PAL08-16 A = GU111927.1; *M. bouillonii* PAL08-16 B = GU182894.1; Shrimp *M. bouillonii* 1A = MT826199.1; Shrimp *M. bouillonii* 1B = MT826200.1; Shrimp *M. bouillonii* 3A = MK299234.1; Shrimp *M. bouillonii* 3B = MK299235.1; NonShrimp *M. bouillonii* 4A = MT826201.1; NonShrimp *M. bouillonii* 4B = MT826202.1; Shrimp *M. bouillonii* 5A = MK299236.1; Shrimp M. bouillonii 5B = MK299237.1; NonShrimp *M. bouillonii* 6A = MT826203.1; NonShrimp *M. bouillonii* 6B = MT826204.1; Shrimp *M. bouillonii* 7A = MT826205.1; Shrimp *M. bouillonii* 7B = MT826206.1; NonShrimp *M. bouillonii* 8A = MT826207.1; NonShrimp *M. bouillonii* 8B = MT826208.1.

**Supplementary Methods – Knowledge Aggregation Questionnaire**

In March of 2017, twenty natural products researchers with previous field experience with *M. bouillonii* were requested to complete a knowledge aggregation questionnaire designed to capture their observations on the distribution and patterns of *M. bouillonii* (See Supplementary Table S6 for questions and tabulation of responses). The majority of scientific literature regarding *M. bouillonii* and *A. frontalis* is focused on the natural products chemistry of *M. bouillonii*, and collecting *M. bouillonii* from the field is a necessity for studying its natural products chemistry, suggesting that the population of natural products researchers responsible for these scholarly efforts could hold previously undocumented insights about *M. bouillonii* and *A. frontalis* colonies in an ecological context. The aggregation of recalled observations was administered via Google Forms. Seventeen responses were received, including those of the authors. Precedent for the use of this researcher questionnaire is provided by prior studies in which fishermen anecdotes were used to gain knowledge of historical fish abundances (Paterson 2010) and the reliance on surveys completed by the Florida dive community to assess the distribution of a blooming cyanobacterium (Paul et al. 2005). In the absence of substantial ecological study of *M. bouillonii* and *A. frontalis*, gaining insights from researchers with experience in collecting *M. bouillonii* and observing it in the field proved to be a useful approach in aggregating collective knowledge of the organisms’ growth patterns and habits. Confidence intervals (95%) were calculated for answers to each question in the questionnaire, based on sample standard deviation using Microsoft Excel. There are limitations to this approach; the researchers who participated in this questionnaire have all previously worked in or with the labs of the study authors, potentially limiting the diversity and independence of perspectives captured by this questionnaire. Additionally, in some cases, participants in the questionnaire had not studied *M. bouillonii* for many years, leading to mistaken recollections about the organism (e.g. several respondents listed Panama, Puerto Rico, or Costa Rico as locations that they studied *M. bouillonii*; these locations are beyond the organism’s native range).

**Supplementary Results – Knowledge Aggregation Questionnaire**

As described above, a total of seventeen researchers with experience studying and collecting *M. bouillonii*, including the authors of this study, submitted answers to a knowledge aggregation questionnaire aimed at gaining a broader spatial and temporal perspective on the interactions of *M. bouillonii* and *A. frontalis* with corals, (Supplementary Table S6). Their responses spanned twenty-four years of direct study of *M. bouillonii* (1993-2017, collectively over 100 years of study) and encompassed observations from the Philippines, Guam, Saipan, Papua New Guinea, Palmyra Atoll, American Samoa, and India’s Lakshadweep Islands. Researchers were asked to rank their ability to identify *M. bouillonii* in the field, on a scale from 1 (no ability) to 5 (optimal ability). Responses ranged from 2 to 5, with a mean of 4.4±1.04 (s.d.) [4.5±0.94 (s.d.), including authors], suggesting a high level of familiarity and perceived competence in the identification of *M. bouillonii* amongst respondents.

Respondents to the questionnaire rated *M. bouillonii* growth on coral reefs based on four different characteristics, all on a scale of 1 to 5. These included the degree to which overgrowth was observed, perceived negative impact, perceived conflict with other organisms, and level of exposure. Together, these researchers reported that *M. bouillonii* has little tendency to overgrow corals (2.23±0.44, 95% C.I.) [2.00±0.44, 95% C.I., including authors], with four respondents emphasizing the growth of *M. bouillonii* as occurring on old coral structures and other hard substrates, five individuals describing *M. bouillonii* as covering or filling holes and crevices in the reef, and five researchers expressing that a good portion of *M. bouillonii* growth occurs through the interior of coral reef structures as opposed to overgrowing structures. Producing a similar distribution of answers (2.15±0.43, 95% C.I.) [2.00±0.44, 95% C.I., including authors], respondents characterized the impact of *M. bouillonii* on corals as not quite benign, but far from deleterious. When asked to characterize the *M. bouillonii*-coral relationship as harmonious (1) or in conflict (5), respondents judged the relationship to be more harmonious than conflicted (2.23±0.40, 95% C.I.) [2.18±0.38, 95% C.I., including authors]. Respondents described *M. bouillonii* as tending to mostly “not come into direct contact with live coral tissue,” to be “opportunistic on the coral reef system, but not harmful,” and as existing “quite readily where corals are not growing.”

There was slightly less agreement by respondents when asked to assess *M. bouillonii* growth as cryptic and recessed (1) versus exposed (5). Answers of 1, 2, 3, and 5 were all given, with a mean of 2.54±0.50 (95% C.I.) [2.41±0.48, 95% C.I.]. This variability is indicative of the array of different growth patterns described by respondents and observed in different locales. When associated with shrimp, *M. bouillonii* is described as growing with a woven, knitted, or cobweb-like appearance that forms mats, tubes, and complex chambers. Without the shrimp, *M. bouillonii* is morphologically characterized as a ‘puff ball’, a ‘hair ball’, or simply as a loose tuft of filaments. One respondent commented on the growth location of filaments not associated with shrimp, describing their tendency to be found dangling beneath overhanging reef structures. Descriptions of *M. bouillonii* growth when associated with shrimp were considerably more diverse; five respondents reported cyanobacterial colonies as growing recessed in or through coral reef structures, and four expressed a tendency of *M. bouillonii* tubes to be found winding around or encircling the base of calcareous structures, while one individual asserted that colonies are occasionally located in exposed areas.

**Supplementary References**

Geneious version 2019.2 created by Biomatters. Available fromhttps://www.geneious.com

Nübel U, Garcia-Pichel F, Muyzer G (1997) PCR primers to amplify 16S rRNA genes from cyanobacteria. Appl Environ Microbiol 63:3327-3332

Paterson B (2010) Integrating fisher knowledge and scientific assessments. Anim Conserv 13:536-537. https://doi.org/10.1111/j.1469-1795.2010.00419.x

Paul VJ, Thacker RW, Banks K, Golubic S (2005) Benthic cyanobacterial bloom impacts the reefs of South Florida (Broward County, USA). Coral Reefs 24:693-697. https://doi.org/10.1007/s00338-005-0061-x

**Supplementary Video S1**

See Supplementary Video S1 file
